# Adverse Events of Latent Tuberculosis Treatment With Isoniazid in People Living With HIV: A Case-Control Study in a Resource-Rich Setting

**DOI:** 10.7759/cureus.41647

**Published:** 2023-07-10

**Authors:** António Carlos Silveira Machado, Cristóvão Figueiredo, Tiago Teixeira, Carlos Azevedo, Joana Fragoso, Sofia Nunes, Daniel Coutinho, Luís Malheiro

**Affiliations:** 1 Medicine, Faculty of Medicine - University of Porto, Porto, PRT; 2 Infectious Diseases, Vila Nova de Gaia/Espinho Hospital Centre, Vila Nova de Gaia, PRT; 3 Medicine, Porto Academic and Clinical Centre, Porto, PRT

**Keywords:** drug-related adverse reactions, drug-related side effects, immunomodulation, isoniazid, hiv, latent tuberculosis

## Abstract

Introduction

Multiple risk factors, such as human immunodeficiency virus (HIV) infection and immunosuppressive therapies, increase the odds of latent tuberculosis infection (LTBI) reactivation and progression to active tuberculosis. A six-to-nine-month preventive treatment with isoniazid (INH) decreases the risk of LTBI reactivation, but its effectiveness can be limited by its long duration and adverse events (AEs), including liver toxicity. Due to comorbidities and polypharmacy, people living with HIV (PLHIV) may be at increased risk of INH-associated AEs. Our study aimed to assess the prevalence of AEs among patients receiving INH treatment for LTBI, to identify risk factors for their occurrence, and to evaluate whether PLHIV have higher odds of developing INH-associated AEs.

Methods

We conducted a single-center retrospective case-control study, including 130 outpatients with LTBI treated with INH between July 2019 and March 2022. Participants who developed AE (cases) were compared to controls, and a subgroup of PLHIV was compared to HIV-negative participants. Demographics, socioeconomic variables, comorbidities, and clinical variables were compared between study groups. Patient data were obtained from institutional electronic medical records, and outcomes were measured at regularly scheduled appointments.

Results

We included 130 participants, of which 54 were PLHIV. The PLHIV subgroup was significantly younger (p = 0.01) and demonstrated significantly higher prevalences of chronic liver disease, previous viral hepatitis, daily alcohol consumption, and intravenous drug use (IDU). One-third of the participants had an AE (45 cases, 34.6%), with liver toxicity being the most common (22.3%). Participants who developed AEs were significantly older (p = 0.030) and had a higher prevalence of economic hardship (p = 0.037), as well as higher scores of the Charlson comorbidity index (p = 0.002) than the controls. INH withdrawal occurred in 17 participants (13.1%) and was mainly associated with liver toxicity (p < 0.01) and gastrointestinal symptoms (p = 0.022). In the adjusted effect model, an age ≥ 65 years, economic hardship, and excessive alcohol consumption were significantly associated with higher odds of AEs, while HIV infection decreased the odds by 68.4% (p = 0.033).

Conclusions

In our study, INH-associated AEs were common, with liver toxicity being the most frequent. Older age, economic hardship, and excessive alcohol consumption increased the odds of INH-associated AEs, while PLHIV had lower odds of developing INH-associated AEs, even when adjusting for other variables in the multivariate analysis. Further studies should be conducted to assess if these results are replicable in a larger population and in different settings.

## Introduction

Between 5% and 10% of individuals infected with *Mycobacterium tuberculosis* develop active disease during the first two to five years following infection. In the remaining, innate immune responses will either eliminate the infection or lead to an asymptomatic phase of latency where the pathogen successfully persists within its host - latent tuberculosis infection (LTBI). About a quarter of the world’s population is included in this last group, in which affected individuals carry live but inactive bacilli, without risk of transmission or evidence of disease activity [1]. Several risk factors increase the probability of LTBI reactivation and progression to active disease. Many of them are related to a state of immunosuppression, most notably infection with the human immunodeficiency virus (HIV), malignancies, and ongoing immunosuppressive therapy, particularly with tumor necrosis factor-alpha (TNF-α) blockers. In these situations, preventive treatment has been proven to reduce the recurrence rate of active tuberculosis by 60-90% [2].

A six-to-nine-month course of isoniazid (INH) is a recommended regimen to treat LTBI [3]. However, adverse events (AE), such as liver toxicity, and long treatment duration, may limit the effectiveness of INH therapy, through poor adherence and early treatment withdrawal. Some studies suggest that people living with HIV (PLHIV) have higher rates of LTBI treatment interruption than people without HIV, but these studies were performed when earlier generations of antiretroviral drugs, known to have unfavorable AEs and drug-drug interactions, were being used [4]. However, it is currently considered that INH can be safely administered in PLHIV, and there is no evidence of added risk of toxicity or drug-drug interactions with current antiretroviral drugs [5].

Although screening for LTBI is recommended in PLHIV and patients prior to initiation of immunosuppression, data comparing the AE profile in these populations are lacking. In our study, we seek to evaluate the prevalence of AEs in patients receiving INH treatment for LTBI in a resource-rich setting, to identify risk factors for their occurrence, and to compare PLHIV being treated for LTBI infection with the HIV-negative population.

## Materials and methods

Population and study design

We performed a retrospective case-control study including patients with LTBI treated with INH in the Infectious Diseases Department at Centro Hospitalar de Vila Nova de Gaia/Espinho (CHVNGE) between July 2019 and March 2022. This study was approved by the CHVNGE ethics committee (reference 13/2023), and a waiver of informed consent was obtained. A sample size of 138 was calculated to have more than 80% power (alpha = 0.05) to detect AEs occurring with a frequency of 30% or greater in our cohort.

Participants were identified by crossing the outpatient clinic lists from the Infectious Diseases Department and the institutional pharmacy database that lists patients who were treated with INH. Participants were included if they fulfilled all of the following criteria: age ≥ 18 years, diagnosis of LTBI as defined by the local protocol, in accordance with national guidelines [6], and an initiated course of six to nine months of INH. LTBI was defined if the patient was asymptomatic, had no evidence of active pulmonary tuberculosis in a chest X-ray or CT-scan, had a reactive interferon-gamma release assay or positive tuberculin skin test, or in the presence of high-risk contact with an active pulmonary or laryngeal tuberculosis case. Patients were also included if they had imagological evidence of previous tuberculosis, without evidence of a complete treatment course, after excluding active disease.

Participants were excluded if they had evidence of active tuberculosis, if treated for LTBI with drugs other than INH, if they had incomplete medical records, in the presence of cirrhosis with a Child-Pugh class B or C, or if they were lost to follow-up. Patients who started INH but suspended treatment at any moment before the established six-nine months were included if the reason for the interruption was known. Patients who developed AEs were compared to patients who did not. Finally, PLHIV were compared to other patients treated for LTBI. As per institutional protocol, pyridoxine was systematically offered simultaneously to INH in PLHIV and undernourished patients.

Data collection

Patient data were obtained from institutional electronic medical records. Discrepancies found in the dataset were evaluated independently by two investigators who cross-checked the medical records and corrected the data. Retrieved data included sociodemographic variables (age, sex, nationality, homeless status, migrant status), economic hardship (as defined per national Social Security standards), HIV-related variables (Centers for Disease Control and Prevention (CDC) HIV staging, HIV treatment regimen), intravenous drug use (IDU), daily alcohol consumption (estimated as drinking more than 30 ml of absolute alcohol or more than two standard drinks per day), personal history of viral hepatitis (chronic viral hepatitis or previously “cured” hepatitis C or B), comorbidities (chronic liver disease, chronic kidney disease, neuropsychiatric disease, and other nonspecific conditions), Charlson comorbidity index (CCI), and hepatotoxic medication (drugs with a probability of liver transaminases elevation considered at least “frequent” or ≥1/100 in the summary of product characteristics). Chronic liver disease was considered if the participant had liver fibrosis (defined as a liver stiffness result of F1 or worse in hepatic elastography) secondary to any current or previous comorbidity, including viral hepatitis, hepatic steatosis, liver alcohol disease, or others. Chronic liver disease was further classified as advanced liver disease if the participant had evidence of advanced liver fibrosis (defined as a liver stiffness result of F3 in hepatic elastography) or evidence of cirrhosis (Child-Pugh classes A, B, and C).

The primary outcome was a composite of any AE developed after the initiation of INH. Secondary outcomes were defined as each of the independent AE. Liver toxicity was defined as grade I (mild) if aspartate aminotransferase (AST) or alanine aminotransferase (ALT) serum levels were 1.25-2.99x the upper limit of normal (ULN), grade 2 (moderate) if 3-4.99x the ULN, grade 3 (severe) if 5.0-19.99x the ULN, or grade 4 (potentially life-threatening) if equal or higher than 20x the ULN. The other independent AEs were neurotoxicity (including peripheral neuropathy), gastrointestinal symptoms, dermatological events, and a separate group encompassing other types of AEs. Treatment completion was also considered a secondary outcome, defined as completing treatment with a six-nine-month INH course, as primarily defined by the treating clinician. Treatment was considered incomplete if a participant refused or ceased treatment earlier for any reason.

Participants were screened for the occurrence of AEs at regularly scheduled appointments that happened at standard intervals of two weeks in the first month, monthly in the second and third months, and then quarterly for the rest of the treatment, through history taking and physical examination, as well as complementary laboratory studies in each programmed appointment.

Statistical analysis

For the descriptive analysis, all categorical data variables were described by their absolute frequencies and proportions (%). Age was categorized as ≥ 65 years, and the CCI score was categorized as ≥ 3. We described the variables and outcomes within the study population as a whole (total) and between PLHIV and other patients. 

For the main objective of this study, cases with composite outcomes were compared to controls. In the univariate analysis, the chi-squared (χ^2^) test was used for the inferential statistical comparison between dichotomic categorical variables. The significance level for all tests was defined as p < 0.05. A multivariate analysis was then performed using logistic regression in which variables with a p-value < 0.10 identified in the univariate analysis were included. Other relevant variables identified while comparing PLHIV with other patients with LTBI were added to the model, if considered clinically relevant. The odds ratio (OR), with a 95% confidence interval (CI), was used to describe the results. We then repeated this analysis for each of the secondary outcomes. However, due to the limited number of cases within each analysis, only the univariate analysis was performed.

Data treatment and analysis were conducted using IBM SPSS Statistics®, software version 27 (IBM Corp., Armonk, NY).

## Results

All potentially eligible patients being treated for LTBI (n = 130) during the recruitment period were included in the study and the ultimate data analysis. The characteristics and outcomes of the study population and differences between the PLHIV and other patients can be found in Table 1. The study population had a median age of 56.5 (Q1-Q3, 49.5-66.0) years, with over 30% of the participants presenting with an age equal to or greater than 65 years. Male sex was slightly predominant, with 73 (56.2%) male participants in the overall study population. While we did not identify any homeless participants, 43% of the total population fulfilled the criteria for economic hardship.

**Table 1:** Descriptive analysis of the study population and univariate analysis of PLHIV and HIV-negative subgroups. PLHIV - People living with HIV

Variables	Total (n=130)	PLHIV (n=54)	HIV-negative (n=76)	p-value
Age ≥ 65 years	40 (30.8%)	11 (20.4%)	29 (38.2%)	0.030
Sex				0.006
Male	73 (56.2%)	38 (70.4%)	35 (46.1%)	
Female	57 (43.8%)	16 (29.6%)	41 (53.9%)	
Homeless				
Migrant	15 (11.5%)	11 (20.4%)	4 (5.3%)	0.008
Economic hardship	56 (43.1%)	24 (44.4%)	32 (42.1%)	0.791
HIV diagnosis	54 (41.5%)			
Intravenous drug use	14 (10.8%)	13 (24.1%)	1 (1.3%)	< 0.001
Daily alcohol consumption	26 (20.0%)	18 (33.3%)	8 (10.5%)	0.001
Previous viral hepatitis	30 (23.1%)	22 (40.7%)	8 (10.5%)	< 0.001
Chronic viral hepatitis	4 (3.1%)	2 (3.7%)	2 (2.6%)	1.000
Any chronic liver disease	26 (20.0%)	22 (40.7%)	4 (5.3%)	< 0.001
Advanced liver disease	6 (4.6%)	6 (11.1%)		0.004
Steatosis	7 (5.4%)	6 (11.1%)	1 (1.3%)	0.02
Hepatotoxic medication	32 (24.6%)	1 (1.9%)	31 (40.8%)	< 0.001
Chronic kidney disease	10 (7.7%)	4 (7.4%)	6 (7.9%)	1.000
Neuropsychiatric conditions	26 (20.0%)	9 (16.7%)	17 (22.4%)	0.423
Charlson comorbidity index score ≥ 3	62 (47.7%)	22 (40.7%)	40 (52.6%)	0.181
Liver toxicity	29 (22.3%)	12 (22.2%)	17 (22.4%)	0.984
Grade 1	16 (12.3%)	9 (16.7%)	7 (9.2%)	
Grade 2	4 (3.1%)	1 (1.9%)	3 (3.9%)	
Grade 3	8 (6.2%)	2 (3.1%)	6 (7.9%)	
Grade 4	1 (0.8%)		1 (1.3%)	
Gastrointestinal adverse events	14 (10.8%)	3 (5.6%)	11 (14.5%)	0.106
Neurological adverse events	6 (4.6%)	2 (3.7%)	4 (5.3%)	1.000
Peripheral neuropathy	4 (3.1%)	1 (1.9%)	3 (3.9%)	0.641
Dermatological adverse events	4 (3.1%)	1 (1.9%)	3 (3.9%)	0.641
Composite outcome	45 (34.6%)	15 (27.8%)	30 (39.5%)	0.167
Need for treatment withdrawal	17 (13.1%)	4 (7.4%)	13 (17.1%)	0.106

A total of 54 (41.5%) participants were PLHIV, of which 12 patients had CDC HIV stage three. Excluding one elite controller, all HIV-positive patients were being treated with antiretroviral therapy (ART), with combined class regimens including a backbone of one or two nucleoside reverse transcriptase inhibitors combined with either a non-nucleoside reverse transcriptase inhibitor, a boosted protease inhibitor, or an integrase inhibitor. 

In the HIV-negative group (n = 76), the main reason for LTBI treatment was the impending treatment of autoimmune disease with an immunosuppressive drug. The main diseases, and corresponding proportion of affected individuals within this subgroup, were systemic rheumatic diseases such as rheumatoid arthritis (34.2%), psoriatic arthritis (5.3%), vasculitis (3.9%), ankylosing spondylitis (3.9%), and systemic lupus erythematosus (2.6%); organ-specific autoimmune diseases such as inflammatory bowel disease (21.1%), interstitial lung disease (10.5%), and multiple sclerosis (2.6%); or hemolytic autoimmune diseases such as paroxysmal nocturnal hemoglobinuria (2.6%). About 14.5% of the patients had other diseases, and two individuals were affected by two concurrent autoimmune diseases. The three most commonly prescribed immunosuppressive drug classes were anti-TNF agents (infliximab, adalimumab, and etanercept) in 19 patients, methotrexate in 16 patients, and rituximab in six patients. Other prescribed drugs included mycophenolate mofetil, cyclophosphamide, leflunomide, tocilizumab, ustekinumab, secukinumab, and eculizumab.

When comparing PLHIV with the HIV-negative subgroup, PLHIV were found to be significantly younger, with a median age of 54 years versus 60.5 years (p = 0.01). PLHIV also had a significantly higher prevalence of chronic liver disease, viral hepatitis, hepatic steatosis, daily alcohol consumption, and IDU. In contrast, chronic use of hepatotoxic medication was more common in the HIV-negative subgroup (Table 1).

Evaluation of outcomes

About one-third of all participants developed at least one AE attributable to INH, with liver toxicity being the most frequent (22.3% of study participants) and dermatological events the rarest (3.1% of study participants). Withdrawal from treatment occurred in 17 participants (13.1%), one of them due to fulminant hepatitis, leading to the patient’s death (Table 1). The main reasons for withdrawal were liver toxicity in eight (6.1%) participants, gastrointestinal intolerance (diarrhea) in three (2.3%) participants, neurological symptoms (dizziness) in two (1.5%) participants, and exanthem in two (1.5%) participants. One participant discontinued treatment due to pill burden, and another participant discontinued treatment against doctors’ order due to fears of side effects after an unplanned pregnancy. Most treatment withdrawals (12 participants, 70.5% of treatment withdrawals) occurred in the first three months after starting INH, and, in eight (47.0%) participants, it occurred in the first month of treatment. Among the participants who interrupted treatment in the first month, the main reason was liver toxicity (five participants, 62.5%). Both cutaneous reactions occurred in the first month. The two patients whose main complaint was diarrhea interrupted treatment after completing three months of daily INH.

Participants with the composite outcome were significantly older, had a higher prevalence of economic hardship, and had higher scores of the CCI (Table 2). Albeit not statistically significant, there was a tendency for a higher frequency of daily alcohol consumption and neuropsychiatric conditions among those with the composite outcome. No other statistically significant differences were found between the composite outcome subgroup and controls. Within the PLHIV group, we did not find any difference in the prevalence of the composite outcome when considering the HIV treatment regimen or CDC HIV staging.

**Table 2:** Univariate analysis comparing the composite outcome group and controls. IDU - Intravenous drug use; CKD - Chronic kidney disease

Variables	Composite outcome (n=45)	Controls (n=85)	p-value
Age ≥ 65 years	20 (44.4%)	20 (23.5%)	0.014
Sex			0.399
Male	23 (51.1%)	50 (58.8%)	
Female	22 (48.9%)	35 (41.2%)	
Migrant	3 (6.7%)	12 (14.1%)	0.206
Economic hardship	25 (55.6%)	31 (36.5%)	0.037
HIV diagnosis	15 (33.3%)	39 (45.9%)	0.167
IDU	5 (11.1%)	9 (10.6%)	1.000
Daily alcohol consumption	13 (28.9%)	13 (15.3%)	0.065
History of viral hepatitis (non-chronic)	9 (20.0%)	21 (24.7%)	0.545
Chronic viral hepatitis	3 (6.7%)	1 (1.2%)	0.119
Any liver disease	11 (24.4%)	15 (17.6%)	0.357
Advanced liver disease	3 (6.7%)	3 (3.5%)	0.416
Steatosis	4 (8.9%)	3 (3.5%)	0.234
Hepatotoxic medication	12 (26.7%)	20 (23.5%)	0.693
CKD	3 (6.7%)	7 (8.2%)	1.000
Neuropsychiatric conditions	13 (28.9%)	13 (15.3%)	0.065
Charlson comorbidity index score ≥ 3	30 (66.7%)	32 (37.6%)	0.002

After adjusting for different variables, the best model predicting the composite outcome included age ≥ 65 years, HIV, economic hardship, daily alcohol consumption, steatosis, and hepatotoxic medication (Table 3). This model predicts that, when adjusting for the mentioned factors, PLHIV have 68.4% less odds of developing INH-associated AEs. On the contrary, an age ≥ 65 years, economic hardship, and daily alcohol consumption all seem to increase the odds of developing the composite outcome, by 2.4, 2.6, and 4.2 times, respectively. The results for steatosis and hepatotoxic medication were not statistically significant after adjusting for other factors.

**Table 3:** Multivariate analysis of the composite outcome. CI - Confidence interval

Variables	Odds ratio	p-value	95% CI for the odds ratio
HIV diagnosis	0.316	0.033	0.110-0.911
Age ≥ 65 years	2.370	0.044	1.025-5.479
Economic hardship	2.636	0.019	1.173-5.922
Daily alcohol consumption	4.241	0.008	1.455-12.356
Steatosis	4.182	0.109	0.727-24.057
Hepatotoxic medication	0.752	0.571	0.281-2.013

A separate univariate analysis was additionally performed for each of the secondary outcomes, including liver toxicity, gastrointestinal AEs, neurotoxicity, dermatological AE, and the need for treatment withdrawal.

Liver toxicity developed in 29 (22.3%) participants. Transient grade 1 (mild) liver toxicity developed in 12.6% of patients and was the most common in both PLHIV and HIV-negative patients (Table 1). Grade 2-4 toxicities were more common in HIV-negative patients. The median timing for liver toxicity was four weeks (Q1-Q3, 2-12 weeks) and varied between two weeks and 32 weeks after starting INH. The median timing for grades 1 and 2 (six and three weeks, respectively) was lower than for grades 3 and 4 (12 and eight weeks, respectively), although the difference was not statistically significant.

Liver toxicity was significantly associated with daily alcohol consumption (p = 0.001), chronic viral hepatitis infection (hepatitis B or C) (p = 0.035), and a CCI score equal to or above three (p = 0.029). No other variables were significantly associated with liver toxicity, although there was a tendency for a higher prevalence of any preexisting liver disease and economic hardship in this group of patients. Gastrointestinal AEs seemed to be significantly more frequent in older patients, with 57.1% of those affected having an age equal to or greater than 65 years, versus only 27.6% in the counterposed group (p = 0.021). The univariate analysis for neurotoxicity and dermatological AE rendered no significant differences between groups. Peripheral neuropathy was more frequent in HIV-negative patients than PLHIV, but the difference was not statistically significant.

The need for treatment withdrawal was more significantly associated with the occurrence of hepatoxicity (p < 0.01) and gastrointestinal AEs (p = 0.022) than other types of adverse effects of treatment.

## Discussion

By comparing patients who developed AEs in the course of their treatment with those who did not, we set out to investigate potential individual factors influencing the likelihood of occurrence of INH-associated AEs in the study population and, more specifically, the effect of HIV infection on the probability of developing an adverse drug reaction.

Currently, studies directly comparing the risk of INH-associated AEs in LTBI treatment between PLHIV and HIV-negative patients in resource-rich countries are lacking as most have been focused on particular groups of patients at risk of LTBI progression [7]. Only a few studies addressing INH efficacy and side effects in LTBI treatment included both PLHIV and other patients, and a recent meta-analysis failed to identify an increased incidence of AEs after stratifying for immunosuppression and HIV status [8].

Advanced age and excessive alcohol intake are well-established risk factors for antituberculosis drug-induced hepatotoxicity and peripheral neuropathy [4]. Due to multiple age-related factors, including multimorbidity, polypharmacy, and changes in drug pharmacokinetics, increasing age seems to represent an important risk factor for adverse drug reactions in general [9]. In economically deprived groups, insufficient income, lower levels of educational attainment, and precarious working and living conditions all contribute to drive and perpetuate health inequalities, which may manifest as worse health outcomes and multimorbidity in poorer groups [10], at least partially explaining our results regarding economic hardship. In this sense, our findings were not surprising, since other studies and general medical knowledge support them.

On the contrary, the reduced risk associated with HIV infection demonstrated in our study was unexpected. Several publications refer to HIV infection as a factor responsible for increased risk of hepatoxicity [11,12] and peripheral neuropathy [4] in patients treated with antituberculosis drugs, although one statement by the American Thoracic Society mentions that PLHIV appear to develop INH-induced liver toxicity in a range comparable to HIV-negative individuals [13]. However, recent data suggest that some HIV treatment regimens may be associated with a higher risk of AE during LTBI treatment with INH combined with rifapentine [14]. Nonetheless, no published evidence supports a real independent risk-reducing role of HIV on INH-related AEs incidence, and, as already mentioned, the few studies stratifying for HIV status failed to find a difference between these two groups [8].

We were unable to exclude unmeasured differences between the study subgroups as the cause of our findings, particularly when considering that the PLHIV subjects were compared to a subgroup mainly composed of participants with autoimmune diseases and a fundamentally distinct clinical profile. Both subgroups were significantly different, which initially led us to anticipate more AEs in the PLHIV subgroup, as they had a higher prevalence of chronic liver disease, viral hepatitis, daily alcohol consumption, economic hardship, and liver steatosis. Age, already mentioned as an important factor for INH-associated AEs, was significantly lower in the PLHIV subgroup, but the risk-reducing effect of HIV was maintained after adjusting for this variable in the multivariate analysis. In addition, we should highlight that the effect of HIV on the primary outcome was demonstrated even when adjusting for comorbidities that are expected to increase INH-associated AE, such as daily alcohol consumption, steatosis, and hepatotoxic medication. We must also consider that the participants with autoimmune diseases, besides having a significantly higher use of hepatotoxic medication, may tend to present with an overall worse clinical and functional status than PLHIV, possibly overestimating the protective effect of HIV on the study outcomes. Furthermore, peripheral neuropathy was more common in HIV-negative patients. While the difference was not statistically significant, it may have contributed to the composite outcome. We believe that the reason for this difference may be due to the routine administration of pyridoxine to PLHIV taking INH as per institutional protocol, in contrast with patients in the other subgroup.

Although toxic metabolites generated during INH metabolization may directly induce liver toxicity, recent literature points out that the mechanism of INH-induced hepatotoxicity may also be immune-mediated [15]. Metushi et al. found an increase in CD4+ T helper (Th) 17 proinflammatory cells coincident with mild liver transaminase elevations in patients treated with INH [16]. Simultaneously, interleukin (IL)-10, an anti-inflammatory cytokine that inhibits Th1 and Th2 immune responses, was also found to increase, suggesting that IL-10+ T cells could induce immune tolerance, preventing the progression of mild liver injury to liver failure [16]. Th17 cells are highly vulnerable to HIV-1 infection, and severe depletion of Th17 cells is documented early in the course of HIV infection. Even after initiation of antiretroviral therapy, recovery of Th17 cells is variable between individuals, and it may only be achieved partially [17]. Furthermore, higher levels of circulating plasma IL-10 have been reported in HIV-infected individuals when compared with healthy controls, with the highest levels detected in patients with more advanced disease stages and higher viremia [18]. It is possible that Th17 cell depletion and increased IL-10 secretion in PLHIV may provide an anti-inflammatory milieu, mitigating the immune-mediated liver injury induced by INH [16]. On the other hand, Th17 cells have been implicated in the pathogenesis of the most common autoimmune diseases, including psoriasis, rheumatoid arthritis, inflammatory bowel disease, and multiple sclerosis, potentially contributing to the findings in our study [19]. Further studies should aim to understand if these differences are present in other settings and the immunological basis behind these findings.

Strong features of our study include the long-term follow-up of participants, which permitted a more sensitive detection of cases over the prolonged treatment course and a low rate of drop-outs. Liver transaminase levels were measured and recorded following a well-defined protocol that minimized missing data bias. Finally, the multivariate analysis allowed us to adjust for important variables when studying the independent effect of predictor variables on the primary outcome.

There are, however, some limitations to our study. The single-center basis, and the selection bias (due to convenience sampling) introduced by the direct comparison of a subgroup of PLHIV with an HIV-negative subgroup almost entirely affected by autoimmune diseases, may compromise the generalizability of our findings. The small number of enrolled participants and the retrospective nature of our study may not allow a full quantification of measured effects, limiting both the internal and external validity of our results. We cannot exclude the possibility of missing participant data, recall bias, and self-report bias when assessing outcomes with an important subjective component (for instance, gastrointestinal symptoms might be differentially perceived and reported by PLHIV and HIV-negative participants). Another limitation is that we did not include a healthy control group to whom we could compare both subgroups, albeit a cohort of “healthy” participants would likely be clinically very different from our subgroups, due to predictable inferior prevalence of comorbidities and younger age, and would probably be clinically managed outside a tertiary care setting.

## Conclusions

INH-associated AEs during LTBI treatment are common and may lead to treatment withdrawal. Liver toxicity is the most common AE and seems to increase with older age, economic hardship, and daily alcohol consumption. When compared to patients with autoimmune diseases treated with INH, well-controlled PLHIV seem to have lower odds of developing INH-associated AEs, even when adjusting for other variables, such as well-known risk factors for INH-associated liver toxicity. Further studies should be conducted to assess if these results are replicable in a larger population and in different settings and to understand the biological basis underlying these findings.
